# The effect of combined Epidural-general Anesthesia on Hemodynamic Instability during Pheochromocytoma and Paraganglioma Surgery: A multicenter retrospective cohort study

**DOI:** 10.7150/ijms.47299

**Published:** 2020-07-19

**Authors:** Soeun Jeon, Ah-Reum Cho, Hyun-Su Ri, Hyeon-Jeong Lee, Jeong-Min Hong, Dowon Lee, Eun Ji Park, Jinsil Kim, Christine Kang

**Affiliations:** 1 Department of Anesthesia and Pain Medicine, Pusan National University, School of Medicine, Yangsan, Republic of Korea.; 2 Department of Anesthesia and Pain Medicine, Biomedical Research Institute, Pusan National University Hospital, Busan, Republic of Korea.; 3 Department of Anesthesia and Pain Medicine, Pusan National University Yangsan Hospital, Yangsan, Republic of Korea.

**Keywords:** analgesia, epidural, hounsfield unit, paraganglioma, pheochromocytoma

## Abstract

**Objectives:** The purpose of this study was to compare the effects of combined epidural-general anesthesia with those of general anesthesia alone on hemodynamic instability (intraoperative hypotension and hypertensive crisis) during pheochromocytoma and sympathetic paraganglioma surgery.

**Methods:** A total of 119 patients' medical records were reviewed who were diagnosed as having pheochromocytoma and sympathetic paraganglioma on the basis of histological findings. Intraoperative hypotension was defined as a mean blood pressure < 60 mmHg or a decrease > 30% in baseline systolic blood pressure after adrenal vein ligation. Hypertensive crisis was defined as a systolic blood pressure > 200 mmHg or an increase > 30% in baseline systolic blood pressure during the operation. The predictor variables for intraoperative hypotension and hypertensive crisis were analyzed with logistic regression models. Data were presented as adjusted odds ratio with 95% confidence interval.

**Results:** The independent predictors of intraoperative hypotension were an increased attenuation number on unenhanced computed tomography (1.112 [1.009-1.226], p = 0.033), a high baseline mean blood pressure (1.063 [1.012-1.117], p = 0.015), and the combined epidural-general anesthesia (5.439 [1.410-20.977], p = 0.014). In contrast, an increased attenuation number on unenhanced computed tomography was the only independent predictor of hypertensive crisis (1.087 [1.021-1.158], p = 0.009).

**Conclusions:** The combined epidural-general anesthesia was not effective in attenuating hypertensive responses, but could have exacerbated intraoperative hypotension. These findings should be taken into account before selecting the anesthetic technique in pheochromocytoma and sympathetic paraganglioma surgery.

## Introduction

Pheochromocytomas and sympathetic paragangliomas (PPGLs) are chromaffin cell tumors that produce, store, and secrete catecholamines 1. The most common symptoms of these two tumors include hypertension, headache, palpitation, and diaphoresis, all of which are caused by catecholamine excess 2. Although the incidence of these tumors is only 0.04-0.57 cases in 100,000 person-years 3, a diagnosis of PPGL is clinically significant. Without treatment, these tumors can lead to lethal complications, but with proper treatment, over 90% of cases are curable 4.

Surgical resection is the treatment of choice for PPGLs 4,5. Recent advances in diagnostic tools, pharmacological management, and surgical and anesthetic techniques have dramatically improved the surgical outcomes of PPGLs 4,6. However, surgical procedures for these catecholamine-secreting tumors are frequently accompanied by hemodynamic instability, which is divided into two sequential phases based on the ligation of the tumor: the hypertensive phase before tumor ligation and the hypotensive phase after tumor ligation 7. The hypertensive crisis has been reported to occur in 51%-85% of PPGL surgeries 8-10. This crisis is caused by excessive catecholamine release during endotracheal intubation, peritoneal insufflation with CO_2_, and tumor manipulation 11,12. Conversely, intraoperative hypotension is known to occur in 44%-77% of PPGL surgeries 9,13. The hypotensive event frequently occurs after tumor resection, and it is caused by intravascular volume depletion, abrupt withdrawal of catecholamine after tumor removal, and chronic downregulation of α and β adrenergic receptors 5,13,14. Either symptom can lead to perioperative morbidity and mortality.

The combined epidural-general anesthesia technique (GE) has been widely used in PPGL resection. This technique has been reported to facilitate hemodynamic stability before tumor isolation and reduce pain and complications after surgery 6,15. However, the sympathectomy-mediated cardiovascular depression associated with an epidural block may exacerbate the intraoperative hypotension and lead to hemodynamic collapse.

The primary purpose of this retrospective cohort study was to compare the effects of GE with those of general anesthesia alone (GA) on hemodynamic instability (intraoperative hypotension and hypertensive crisis) during PPGL surgery. We also investigated the risk factors for predicting hemodynamic instability in PPGL surgery.

## Methods

### Study design and subjects

The institutional review boards of Pusan National University Hospital and Pusan National University Yangsan Hospital designated this retrospective cohort study as exempt (ID: H-1903-022-077 and 05-2019-055). The subjects of this study were patients who had been histologically diagnosed with PPGLs from January 2000 to December 2018 at Pusan National University Hospital or Pusan National University Yangsan Hospital. The following patients were excluded from the study: those with duplicated data, patients younger than 18 years, patients with head and neck paraganglioma, patients who underwent co-operative surgery, patients who had not undergone general anesthesia, and patients with more than 10% missing values.

### Anesthetic management

In the operating room, standard (electrocardiography, pulse oximetry, noninvasive blood pressure [NIBP] measurement, esophageal stethoscope temperature), depth of anesthesia (entropy or bispectral index measurement), and intra-arterial blood pressure monitoring were performed. A central or large-bore peripheral intravenous catheter was inserted in all patients.

Selection of anesthetic agents and techniques was made entirely by the attending anesthesiologists. In patients who underwent GE techniques, the epidural catheter was inserted into the lower thoracic epidural space (T8-12) according to the surgical incision site prior to general anesthesia induction. To provide adequate analgesia during surgery, local anesthetics (0.2% ropivacaine or 0.2% chirocaine) and supplemental opioid (3 mg of morphine sulfate or 50 mcg of fentanyl) were epidurally administered, and intravenous remifentanil infusion was occasionally added. The administration of the epidural loading dose was completed within 30 minutes after anesthesia induction, and no epidural medication was administered during the main surgical procedure. In patients undergoing GA techniques, intravenous opioid administration (remifentanil infusion or fentanyl bolus) was used for analgesia during induction and maintenance of anesthesia.

Induction of general anesthesia was performed using an intravenous sedative hypnotic agent (thiopental sodium, propofol, or etomidate) and muscle relaxants (succinylcholine, cisatracurium, or rocuronium). Anesthesia was maintained with continuous administration of sevoflurane, desflurane, isoflurane, or propofol, along with intermittent boluses of nondepolarizing muscle relaxants (vecuronium, cisatracurium, or rocuronium).

### Assessment of outcomes

Intraoperative hypotension and hypertensive crisis were defined before data collection and analysis. Intraoperative hypotension was defined as a mean intra-arterial blood pressure < 60 mmHg or a systolic intra-arterial blood pressure (SBP) reduction > 30% immediately before induction of anesthesia (SBPb) during the operation. Hypertensive crisis was defined by an SBP > 200 mmHg or an increase of > 30% in the SBPb during the operation.

The following data were extracted from the electronic medical records: (1) baseline patient characteristics—age, sex, height, weight, clinical manifestations, American Society of Anesthesiologists (ASA) classification, left ventricular ejection fraction (LVEF), comorbidities; (2) preoperative computed tomography (CT) findings—The largest tumor diameter in the transverse plane and mean attenuation (Hounsfield unit: HU) on unenhanced CT. The mean attenuation number of the PPGL mass was measured using a circular region-of-interest (ROI) cursor. HU was measured in the central area while avoiding periadrenal retroperitoneal fat, and the mean HU value measured in two continuous sections was used; (3) preoperative catecholamine levels— plasma norepinephrine (pNE), plasma epinephrine (pEpi), urine vanillylmandelic acid (uVMA), and urine metanephrine (uMN) levels; (4) premedications— use of α-blocker and duration; (5) intraoperative data— anesthetic technique (GE vs. GA), induction and maintenance agents, use of remifentanil, method of surgery (laparoscopic or open), operation time, estimated blood loss, amount of fluid administration, urine output, and blood transfusion; (6) hemodynamic data—mean blood pressure before premedication (MBPpre), mean blood pressure immediately before induction of anesthesia (MBPb), and hemodynamic values during general anesthesia. Variables with data missing for more than 30% of the patients (plasma metanephrine, plasma normetanephrine, urine norepinephrine, urine epinephrine, and urine normetanephrine) were excluded from the analysis.

### Statistical analysis

All analyses were performed using IBM SPSS Statistics (version 22; IBM Corporation, Armonk, NY) and MedCalc (version 18.11.6; MedCalc Software bvba, Ostend, Belgium). Continuous variables were presented as mean ± standard deviation (SD) or as median and interquartile range (IQR). Categorical data were reported as absolute numbers and percentages. The results of binary logistic regression analyses were presented as odds ratio (OR) or adjusted OR with 95% confidence interval (95% CI).

Patient data were grouped according to anesthetic technique (GE vs. GA) and the occurrence of hemodynamic instability to compare preoperative patient demographics, tumor characteristics, and intraoperative variables. After the normality test, normally distributed data were analyzed using an independent t-test and non-parametric data were analyzed using the Mann-Whitney U test; categorical data were analyzed using the chi-squared test (with Yates' continuity correction for the 2 × 2 contingency table) or Fisher's exact test.

First, univariate binary logistic regression analysis was performed to evaluate the association between predictor variables and outcome variables (intraoperative hypotension and hypertensive crisis). The predictor variables entered in the univariate analysis were baseline patient characteristics, preoperative CT findings, preoperative catecholamine level, premedications, intraoperative data, and preoperative hemodynamic data (MBPpre and MBPb).

The candidate predictors entered in the multivariate binary logistic regression analysis were predictor variables with p values less than 0.25 in the univariate analysis. The anesthetic technique (GE vs. GA) was also entered in the multivariate binary logistic regression analysis regardless of p value because it was our primary predictor variable. Predictor variable selection was performed by the backward elimination method based on the probability of the likelihood-ratio statistic, with p value ≥ 0.1 as the removal criterion. Patients with missing data for one or more predictors were excluded from the logistic regression analysis. To evaluate the usefulness and goodness-of-fit of the multivariate logistic regression model, Nagelkerke R^2^ and the Hosmer-Lemeshow chi-squared statistics were calculated. The probability of each case was calculated and the area under the receiver operating characteristic curve (AUROC) was determined to evaluate the discriminative power of the multivariate logistic regression models; AUROC was also used to determine the optimal cut-off values for continuous predictor variables.

## Results

**Figure 1:**
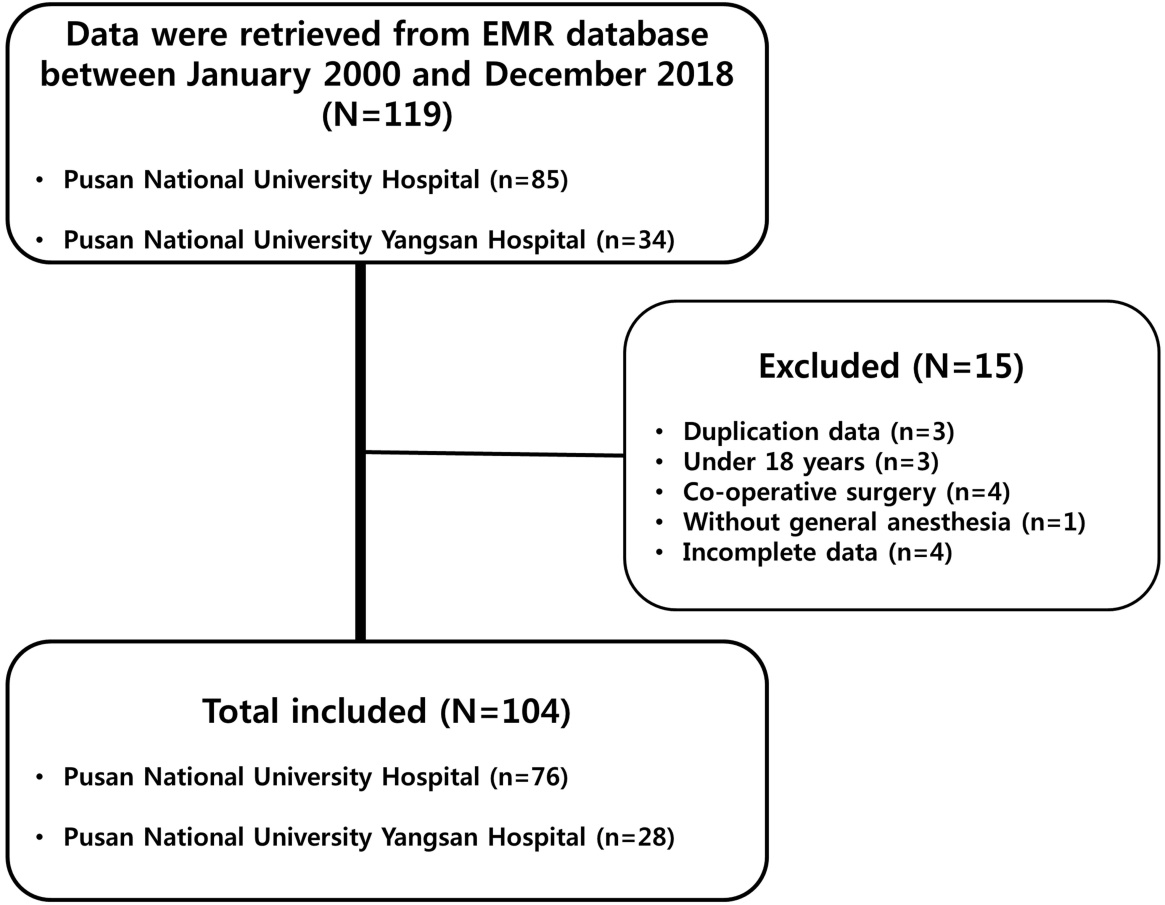
Study flow chart. Of the 119 patients' records retrieved, 15 were excluded for the following reasons: 3 due to duplication, 3 due to patients' age under 18 years old, 4 due to patients underwent co-operative surgery, 1 due to procedures without general anesthesia, and 4 due to missing values. Electronic medical record (EMR).

Of the 119 patient records retrieved, 15 were excluded for the following reasons: duplication, three cases; patient age under 18 years, three cases; co-operative surgery, four cases; procedures without general anesthesia, one case; and missing values, four cases (Figure 1). Of the 104 patients included in the final sample, 53 (51.0%) underwent surgery with the GE technique, while the remaining 51 patients (49.0%) underwent surgery with the GA technique. The two groups were comparable in terms of preoperative patient demographics, tumor characteristics, and intraoperative variables. In addition, the two groups did not differ in terms of preoperative hemodynamic parameters (Table 1).

### Predictors of intraopertive hypotension

Preoperative patient demographics, tumor characteristics, and intraoperative variables in the groups stratified according to the presence of hypotension after PPGL resection are summarized in Table 2. Of all 104 patients included in this study, intraoperative hypotension occurred in 80 (76.9%) patients. There were significant differences in the baseline BP and anesthetic technique between the two groups.

Univariate analysis presented 6 candidate predictors that might be independently associated with the occurrence of intraoperative hypotension (Table 3). The following six candidate predictors were entered in the multivariate logistic regression analysis: HU value on unenhanced CT, MBPpre, MBPb, α-blocker premedication, duration of pretreatment, and anesthetic technique. The results of variable selection using the backward elimination method showed that the following variables were independent predictors of intraoperative hypotension: increased HU value on unenhanced CT (adjusted OR [95% CI]: 1.112 [1.009-1.226], p = 0.033), high MBPb (adjusted OR [95% CI]: 1.063 [1.012-1.117], p = 0.015), and GE technique (adjusted OR [95% CI]: 5.439 [1.410-20.977], p = 0.014). α-Blocker premedication was included in the final model, but it was not statistically significant (adjusted OR [95% CI]: 5.459 [0.937-31.805], p = 0.059; Table 4). The Hosmer-Lemeshow chi-square statistic, Nagelkerke R^2^, and AUROC of the final model were 4.957 (p = 0.762), 0.403, and 0.852 (95% CI: 0.765-0.938), respectively. The receiver operating characteristic (ROC) analysis to determine the cut-off values of continuous predictor variables showed that the optimal cut-off HU value on unenhanced CT and MBPb were 37 HU (AUROC [95% CI]: 0.654 [0.539-0.757]) and 93.7 mmHg (AUROC [95% CI]: 0.741 [0.646-0.822]).

### Predictors of hypertensive crisis

Preoperative patient demographics, tumor characteristics, and intraoperative variables of the groups stratified according to the occurrence of hypertensive crisis during PPGL resection are summarized in Table 2; hypertensive crises occurred in 57 (54.8%) patients. There were significant differences in age, HU value on unenhanced CT, and tumor diameter between the two groups.

Seven candidate predictors (Table 3) and anesthetic technique were entered in the multivariate logistic regression analysis. The results of variable selection using the backward elimination method showed that an increased HU value on unenhanced CT was the only independent predictor for the occurrence of hypertensive crisis (adjusted OR [95% CI]: 1.087 [1.021-1.158], p = 0.009). Dysarrhythmia (adjusted OR [95% CI]: 0.026 [0.032-1.318], p = 0.095) and GE technique (adjusted OR [95% CI]: 0.378 [0.141-1.015], p = 0.053) were included in the final model, but were not statistically significant (Table 4). The Hosmer-Lemeshow chi-squared statistic, Nagelkerke R^2^, and AUROC of the final model were 6.010 (p = 0.646), 0.198, and 0.727 (95% CI: 0.616, 0.838), respectively. The ROC analysis to determine the cut-off value of the continuous predictor variable showed that the optimal cut-off HU value on unenhanced CT was 36 HU (AUROC [95% CI]: 0.667 [0.553-0.768]).

## Discussion

In this retrospective chart review study, intraoperative hypotension was observed in 80 (76.9%) patients during PPGL resection. The independent predictors of intraoperative hypotension were an increased HU value on unenhanced CT, a high MBPb, and the GE technique. Hypertensive crisis occurred in 57 (54.8%) patients during PPGL resection. The only independent predictor of a hypertensive crisis was an increased HU value on unenhanced CT.

The known risk factors for hemodynamic instability during PPGL surgery are increased preoperative urinary catecholamine levels, larger tumor size, and absence of the α-blocker premedication 1,8,13,16. Previous studies have demonstrated the usefulness of the HU value on unenhanced CT as a diagnostic tool for PPGLs 17. However, to date, no studies have been conducted on the associations between intraoperative hemodynamic instability during PPGLs surgery and HU value on CT scans. In the present study, an increased HU value on unenhanced CT was found to be associated with the occurrence of both intraoperative hypotension and hypertensive crisis during surgical resection of PPGL. The underlying mechanism of this association is elucidative; however, several studies showed that the increased urinary MN or normetanephrine significantly associated with an increased CT attenuation in PPGLs 18,19.

In the present study, a high MBPb was found to be associated with the occurrence of intraoperative hypotension, but not with hypertensive crisis during surgery. Before the resection of PPGLs, blood pressure should be optimized whenever possible, since uncontrolled preoperative hypertension in PPGL patients is known to increase the risk of perioperative complications 20. PPGLs can induce and exacerbate arterial stiffness, myocardial hypertrophy and fibrosis, and these cardiovascular remodeling have been reported to be associated with high blood pressure 21-24. These hypertension-associated morphofunctional changes impair the ability of blood pressure regulation in stressful situations 25,26, which is thought to contribute to the development of intraoperative hypotension during PPGL resection. According to the current recommendations based on recent research, preoperative blood pressure control for PPGL patients is strict; blood pressure should be below 130/85 mmHg (MBP 100 mmHg) in the seated position, while SBP should exceed 90 mmHg in the standing position 5. In the present study, the cut-off value of MBPb to predict intraoperative hypotension was 93.7 mmHg, which is consistent with the aforementioned recommendation.

In the present study, the GE technique was found to be associated with the occurrence of intraoperative hypotension during PPGL surgery. Epidural analgesia is frequently used in combination with general anesthesia in PPGL surgery for the attenuation of intraoperative hemodynamic response and postoperative pain control. Luo et al. 6 demonstrated that, as compared to the GA technique for PPGL resection, the GE technique has a vasodilator-sparing effect by reducing systemic vascular resistance before tumor removal. Furthermore, Li et al. 15 reported that although the GE technique failed to reduce the occurrence of intraoperative hemodynamic fluctuations, it effectively reduced postoperative complications in patients undergoing open surgery for pheochromocytoma. Our results suggest that the GE technique was not effective in preventing hypertensive crisis during PPGL surgery, but it may have exacerbated intraoperative hypotension. This may be due to the fact that the epidural block was not sufficient to inhibit the catecholamine surge during tumor manipulation but exaggerated the sympathectomy and vasodilatory effects after tumor ligation 2,5,27.

Tumor manipulation is the most powerful factor to induce a catecholamine surge during PPGL surgery 12,14,28. Other risk factors previously found to be potentially associated with hypertensive crises include increased preoperative catecholamine level, larger tumor size, and absence of α-blocker premedication 1,5,8,16. However, in the present study, these factors were not found to be associated with hypertensive crisis during PPGL surgery. This controversial outcome may be due to the most powerful factor, surgical manipulation of PPGL, masking the effects of other factors on hypertensive crisis. The surgeon's skill and competence levels are expected to have a significant impact on minimizing tumor manipulation; however, we could not evaluate the impact of this factor, which is a limitation of the present study.

The present study has several other limitations. First, the intraoperative hemodynamic data used in the present study were extracted from electronic anesthetic records. Since these measurements are recorded every 5 minutes, the accuracy of the hemodynamic parameters could have been compromised. Second, due to the low prevalence rate of PPGLs, the recruitment period was over 18 years, and the development of surgical and anesthetic skills may have produced a bias. Finally, due to the low frequency of complications, we could not analyze the correlation between intraoperative hemodynamic instability and the patient's clinical outcome.

## Conclusions

In conclusion, in the present study, an increased HU value on unenhanced CT, a high MBPb, and the GE anesthetic technique were found to be independent predictors of intraoperative hypotension, while an increased HU value on unenhanced CT was found to be an independent predictor of hypertensive crisis during PPGL surgery. Since the GE anesthetic technique has the potential to cause intraoperative hypotension, anesthesiologists should consider these findings before selecting an anesthetic technique in PPGL surgery.

## Figures and Tables

**Table 1:** Patient demographics, tumor characteristics, and intraoperative variables according to anesthetic technique

Variables	Anesthetic techniques		P value
	GA (n=51)	GE (n=53)	
Age (yr)	49.9 (14.8)	48.8 (13.0)	0.685
Sex (Male)	26 (51.0)	30 (56.6)	0.705
**ASA classification**			0.297
I	7 (13.7)	5 (9.4)	
II	36 (70.6)	44 (83.0)	
III	8 (15.7)	4 (7.5)	
**Height (cm)**	163.8 (8.6)	164.5 (7.9)	0.694
**Weight (kg)**	62.0 (9.8)	63.5 (12.1)	0.487
**BMI (kg/m^2^)**	23.0 (3.0)	23.3 (3.5)	0.628
**Preoperative LVEF (%)**	63.0 (60.0-65.5)	64.0 (60.0-65.5)	0.694
**Clinical manifestations**	31 (60.8)	37 (69.8)	0.447
**Comorbidity**			
Stroke	5 (9.8)	1 (1.9)	0.109
Dysarrhythmia	6 (11.8)	2 (3.8)	0.157
Hyperlipidemia	2 (3.9)	3 (5.7)	1.000
Diabetes mellitus	14 (27.5)	20 (37.7)	0.364
**Preoperative CT findings**			
HU on unenhanced CT	33.9 (7.4)	34.0 (9.2)	0.963
Tumor diameter (cm)	6.0 (3.6-36.0)	5.3 (3.6-8.0)	0.474
**Preoperative catecholamine levels**			
Plasma norepinephrine (pg/ml)	343.2 (82.0-947.6)	342.0 (11.5-878.8)	0.872
Plasma epinephrine (pg/ml)	31.8 (0.5-176.3)	72.4 (6.1-151.3)	0.716
Urine vanillylmandelic acid (mg/day)	10.0 (6.0-20.9)	9.1 (6.0-17.5)	0.523
Urine metanephrine (µg/day)	2029.0 (901.5-6993.5)	3700.0 (353.7-3700.0)	0.250
**Preoperative MBP (mmHg)**			
Before premedication	98.5 (93.3-111.2)	100.0 (92.8-110.0)	0.829
Before induction of anesthesia	96.7 (86.7-109.7)	102.0 (90.0-114.7)	0.366
**Premedication**			
α-blocker	46 (90.2)	47 (88.7)	1.000
Duration of pretreatment (days)	30.0 (15.0-43.0)	28.0 (20.3-45.8)	0.966
***Intraoperative data***			
**Induction agent**			0.602
Thiopental	15 (29.4)	18 (34.0)	
Propofol	36 (70.6)	34 (64.2)	
Etomidate	0 (0.0)	1 (1.9)	
**Maintenance agent**			0.614
Inhalation	49 (96.1)	52 (98.1)	
Propofol	2 (3.9)	1 (1.9)	
**Remifentanil**	47 (92.2)	30 (56.6)	< 0.001
**Methods of surgery**			0.324
Open	11 (21.6)	17 (32.1)	
Laparoscopic	40 (78.4)	36 (67.9)	
**Intraoperative hypotension**	33 (64.7)	47 (88.7)	0.008
**Hypertensive crisis**	30 (58.8)	27 (50.9)	0.542

Data were presented as mean (SD), median (IQR), and absolute numbers (%). Combined epidural-general anesthesia technique (GE), general anesthesia alone (GA), American society of anesthesiologists (ASA), body mass index (BMI), left ventricular ejection fraction (LVEF), computed tomography (CT), Hounsfield unit (HU), mean blood pressure (MBP).

**Table 2:** Patient demographics, tumor characteristics, and intraoperative variables according to the occurrence of intraoperative hypotension and hypertensive crisis

Variables	Intraoperative hypotension			Hypertensive crisis		
	No. (n = 24)	Yes (n = 80)	P value	No. (n = 47)	Yes (n = 57)	P value
Age (yr)	49.3 (12.1)	49.3 (14.4)	0.998	52.7 (13.3)	46.5 (13.8)	0.022
Sex (Male)	13 (54.2)	43 (53.8)	1.000	25 (53.2)	31 (54.4)	1.000
**ASA classification**			0.149			0.385
I	5 (20.8)	7 (8.8)		3 (6.4)	9 (15.8)	
II	15 (62.5)	65 (81.3)		38 (30.9)	42 (73.7)	
III	4 (16.7)	8 (10.0)		6 (12.8)	6 (10.5)	
Height (cm)	163.6 (5.9)	164.3 (8.8)	0.655	163.5 (9.0)	164.7 (7.6)	0.464
Weight (kg)	63.6 (9.1)	62.6 (11.5)	0.674	63.9 (13.0)	61.9 (9.0)	0.378
BMI (kg/m^2^)	23.6 (3.3)	23.0 (3.2)	0.450	23.5 (3.4)	22.8 (3.1)	0.298
Preoperative LVEF (%)	63.0 (61.0-65.0)	63.0 (60.0-66.0)	0.779	62.0 (58.8-65.0)	65.0 (60.0-67.5)	0.077
Clinical manifestations	15 (62.5)	53 (66.3)	0.925	28 (59.6)	40 (70.2)	0.356
**Comorbidity**						
**Stroke**	1 (4.2)	5 (6.3)	1.000	2 (4.3)	4 (7.0)	0.687
Dysarrhythmia	1 (4.2)	7 (8.8)	0.678	6 (12.8)	2 (3.5)	0.136
Hyperlipidemia	1 (4.2)	4 (5.0)	1.000	2 (4.3)	3 (5.3)	1.000
Diabetes mellitus	7 (29.2)	27 (33.8)	0.864	17 (36.2)	17 (29.8)	0.634
**Preoperative CT findings**						
HU on unenhanced CT	30.9 (5.7)	35.0 (8.7)	0.055	31.5 (7.9)	36.3 (7.9)	0.009
Tumor diameter (cm)	5.0 (2.8-45.7)	5.6 (3.8-13.5)	0.852	8.0 (4.0-36.0)	4.8 (3.4-8.0)	0.050
**Preoperative catecholamine levels**						
Plasma norepinephrine (pg/ml)	350.6 (121.3-749.8)	338.8 (2.0-1004.7)	0.866	358.0 (128.0- 946.0)	326.3 (0.8-897.0)	0.364
Plasma epinephrine (pg/ml)	35.4 (18.8-181.6)	40.4 (0.4-154.5)	0.679	40.0 (10.6-164.8)	36.1 (0.5-172.1)	0.863
Urine vanillylmandelic acid (mg/day)	9.9 (5.2-20.4)	9.5 (6.6-20.5)	0.609	9.2 (6.4-18.7)	10.4 (5.5-21.9)	0.847
Urine metanephrine (µg/day)	1662.3 (866.7-6655.3)	3100.5 (1099.4-6705.0)	0.433	2585.0 (953.8-4954.8)	3686.0 (930.0-8303.2)	0.317
**Preoperative MBP (mmHg)**						
Before premedication	96.7 (88.3-105.0)	100.0 (93.3-111.7)	0.186	100.0 (92.3-109.9)	98.5 (93.3-113.7)	0.608
Before induction of anesthesia	88.4 (85.4-93.3)	106.3 (97.8-117.5)	<0.001	106.3 (90.0-110.3)	95.0 (86.7-117.5)	0.311
**Premedication**						
α-blocker	19 (79.2)	74 (92.5)	0.121	44 (93.6)	49 (86.0)	0.337
Duration of pretreatment (days)	25.50 (9.25-40.00)	30.00 (21.00-46.00)	0.137	32.0 (23.8-50.0)	27.0 (11.0-42.0)	0.063
***Intraoperative data***						
**Induction agent**			0.083			0.592
Thiopental	10 (41.7)	23 (28.7)		16 (34.0)	17 (29.8)	
Propofol	13 (54.2)	57 (71.3)		30 (63.8)	40 (70.2)	
Etomidate	1 (4.2)	0 (0.0)		1 (2.1)	0 (0.0)	
**Maintenance agent**			1.000			1.000
Inhalation	23 (95.8)	78 (97.5)		46 (97.9)	55 (96.5)	
Propofol	1 (4.2)	2 (2.5)		1 (2.1)	2 (3.5)	
**Remifentanil**	18 (75.0)	59 (73.8)	1.000	35 (74.5)	42 (73.7)	1.000
**Anesthetic technique**			0.008			0.542
General anesthesia alone	18 (75.0)	33 (41.3)		21 (44.7)	30 (52.6)	
Combined epidural-general	6 (25.0)	47 (58.8)		26 (55.3)	27 (47.4)	
**Methods of surgery**			1.000			0.412
Open	5 (20.8)	23 (28.7)		15 (31.9)	13 (22.8)	
Laparoscopic	19 (79.2)	57 (71.3)		32 (68.1)	44 (77.2)	
Operation time (min)	212.5 (168.8-296.3)	195.0 (150.0-247.5)	0.324	195.0 (150.0-240.0)	210.0 (165.0-270.0)	0.294
Amount of fluid administration (ml)	1900.0 (1400.0-3300.0)	2300.0 (1800.0-3800.0)	0.143	2200 (1600-2900)	2200 (1700-4100)	0.311
EBL (ml)	300.0 (100.0-500.0)	300.0 (100.0-500.0)	0.768	200.0 (100.0-500.0)	300.0 (100.0-500.0)	0.315
Urine output (ml)	300.0 (150.0-800.0)	400.0 (200.00-900.000)	0.247	400.0 (200.0-630.0)	400.0 (200.0-1100.0)	0.926
Transfusion	2 (8.3)	20 (25.0)	0.142	8 (17.0)	14 (24.6)	0.487
Hypertensive crisis	15 (62.5)	42 (52.5)	0.529	38 (80.9)	42 (73.7)	0.529

Data were presented as mean (SD), median (IQR), and absolute numbers (%). American society of anesthesiologists (ASA), body mass index (BMI), left ventricular ejection fraction (LVEF), computed tomography (CT), Hounsfield unit (HU), mean blood pressure (MBP).

**Table 3:** Univariate analysis of variables associated with intraoperative hypotension and hypertensive crisis

Variable	Intraoperative hypotension		Hypertensive crisis	
	Unadjusted OR (95% CI)	P value	Unadjusted OR (95% CI)	P value
Age (yr)	1.000 (0.967-1.034)	0.998	0.966 (0.938-0.996)	0.025
Female gender (Ref. Male)	1.017 (0.407-2.540)	0.971	0.953 (0.439-2.068)	0.903
ASA classification	1.265 (0.488-3.282)	0.628	0.597 (0.260-1.370)	0.224
BMI (kg/m^2^)	0.945 (0.817-1.093)	0.446	0.936 (0.827-1.060)	0.297
Preoperative LVEF (%)	0.982 (0.908-1.062)	0.649	1.038 (0.976-1.104)	0.230
Clinical manifestations (Ref. No)	1.178 (0.457-3.038)	0.735	1.597 (0.708-3.601)	0.259
**Comorbidity**				
Stroke (Ref. No)	1.533 (0.170-13.801)	0.703	1.698 (0.297-9.706)	0.552
Dysarrhythmia (Ref. No)	2.205 (0.258-18.878)	0.470	0.248 (0.048-1.295)	0.098
Hyperlipidemia (Ref. No)	1.211 (0.129-11.375)	0.867	1.250 (0.200-7.811)	0.811
Diabetes mellitus (Ref. No)	1.237 (0.458-3.345)	0.675	0.750 (0.330-1.707)	0.493
**Preoperative CT findings**				
HU on unenhanced CT	1.068 (0.997-1.144)	0.059	1.081 (1.017-1.149)	0.012
Tumor diameter (cm)	0.992 (0.974-1.009)	0.344	0.988 (0.972-1.005)	0.168
**Preoperative catecholamine levels**				
Plasma norepinephrine (pg/ml)	1.000 (1.000-1.000)	0.524	1.000 (1.000-1.000)	0.345
Plasma epinephrine (pg/ml)	1.001 (0.999-1.003)	0.473	1.000 (1.000-1.000)	0.486
Urine vanillylmandelic acid (mg/day)	1.007 (0.961-1.054)	0.773	1.012 (0.976-1.050)	0.506
Urine metanephrine (µg/day)	1.000 (1.000-1.000)	0.604	1.000 (1.000-1.000)	0.563
**Preoperative MBP (mmHg)**				
Before premedication	1.024 (0.995-1.054)	0.110	1.013 (0.991-1.035)	0.265
Before induction of anesthesia	1.073 (1.029-1.118)	0.001	0.991 (0.968-1.014)	0.420
**Premedication**				
α-blocker (Ref. No)	3.246 (0.894-11.784)	0.074	0.418 (0.104-1.673)	0.218
Duration of pretreatment (day)	1.012 (0.992-1.033)	0.233	0.999 (0.995-1.003)	0.551
**Intraoperative data**				
Open surgery (Ref. laparoscopic)	1.533 (0.512-4.596)	0.445	0.630 (0.264-1.506)	0.299
Operation time (min)	0.998 (0.994-1.002)	0.305	1.002 (0.998-1.005)	0.370
Inhalation agent (Ref. Propofol)	0.590 (0.051-6.801)	0.672	1.673 (0.147-19.042)	0.678
Remifentanil (Ref. No)	0.937 (0.328-2.675)	0.903	0.960 (0.398-2.318)	0.928
GE technique (Ref. GA technique)	4.273 (1.532-11.915)	0.006	0.727 (0.335-1.578)	0.420
**Hypertensive crisis (Ref. No)**	0.663 (0.260-1.690)	0.390		
**Intraoperative hypotension (Ref. No)**			0.663 (0.260-1.690)	0.390

Reference value (Ref.), American society of anesthesiologists (ASA), body mass index (BMI), left ventricular ejection fraction (LVEF), computed tomography (CT), Hounsfield unit (HU), mean blood pressure (MBP), combined epidural-general anesthesia technique (GE), and general anesthesia alone (GA).

**Table 4:** Multivariate analysis of variables associated with intraoperative hypotension and hypertensive crisis

Variable	Adjusted OR* (95% CI)	P value
**Intraoperative hypotension**		
HU on unenhanced CT	1.112 (1.009-1.226)	0.033
MBPb (mmHg)	1.063 (1.012-1.117)	0.015
α-blocker (Ref. No)	5.459 (0.937-31.805)	0.059
GE techniques (Ref. GA technique)	5.439 (1.410-20.977)	0.014
**Hypertensive crisis**		
Dysarrhythmia (Ref. No)	0.206 (0.032-1.318)	0.095
HU on unenhanced CT	1.087 (1.021-1.158)	0.009
GE techniques (Ref. GA technique)	0.378 (0.141-1.015)	0.053

*Each OR is adjusted for all other variables in the table. Hounsfield unit (HU), computed tomography (CT), mean blood pressure immediately before induction of anesthesia (MBPb), reference value (Ref.), combined epidural-general anesthesia technique (GE), and general anesthesia alone (GA).

## Abbreviations

95% CI: 95% confidence interval
ASA: American Society of Anesthesiologists
AUROC: Area under the receiver operating characteristic curve
CT: Computed tomography
GA: General anesthesia alone
GE: Combined epidural-general anesthesia technique
HU: Hounsfield unit
IQR: Interquartile range
MBPpre: Mean blood pressure before premedication
MBPb: Mean blood pressure immediately before induction of anesthesia
OR: Odds ratio
pEpi: Plasma epinephrine
pNE: Plasma norepinephrine
PPGLs: Pheochromocytomas and sympathetic paragangliomas
SBP: Systolic blood pressure
SBPb: Systolic blood pressure immediately before induction of anesthesia
SD: Standard deviation
uMN: Urine metanephrine
uVMA: Urine vanillylmandelic acid
